# Cook County Hospital

**Published:** 1885-10

**Authors:** Frank S. Johnson


### HOSPITAL REPORTS.

### COOK COUNTY HOSPITAL.

A SUMMARY OF THE FORTY-ONE CASES OF TYPHOID FEVER TREATED IN COOK COUNTY HOSPITAL DURING THE MONTHS OF JULY, AUGUST AND SEPTEMBER, 1883.* *Reported by* FRANK S. JOHNSON, M. D.

Nearly all of these patients were from our foreign population. Their nationalities were :

Swedish, 9; Norwegians, 2; Danish, 1; German, 14; Bohemian, 1; Swiss, 1; Polish, 1; English, 1; Irish, 6; American, 4; not recorded, 1.

Very many of these patients were recent arrivals in this country, and were without homes. It would, therefore, not be right to infer from this list that greater prevalence of this disease exists among foreigners than among the native born. It simply shows a greater number of homeless sick among that class. A similar preponderance of foreigners will be found sick of almost all other diseases treated in this hospital.

The ratio of the sexes does not conform to that usually observed. Twenty-eight cases were males, twelve were females.

In one case no record of the sex was made.

This unusual predominance of the disease among males cannot be taken as the proportion of males to females attacked in our community during that season, for the number of males in the hospital is constantly more than double that of the females, and naturally so, because there are more homeless

* Cases still under treatment on October 1st are not included.

men than women in a community to which there is great immigration. This fact detracts from the value of any conclusions as to the frequency of disease among either sex, based upon the records of this hospital.

Their ages were recorded in thirty-six of the forty-one cases. Of these thirty-six cases, twenty-two were between the ages of eighteen and twenty-five. Two were under eighteen years. The others were all under thirty-five, except one, an old lady of sixty-seven years, with malarial complication.

The mode of onset, as recorded, may, in most cases, be regarded as fairly accurate, but the value of the conclusions is somewhat lessened by the conditions attending the obtaining of the data, for on admission to the hospital, some of the patients were very dull, and their statements were unsatisfactory.

In four of the forty-one cases no history of the onset was obtainable. In thirty-seven cases a history of the early stages was elicited. In seven of these there was well-marked chill; of these, one was a mild case. Three were moderately severe cases, two were severe cases and one was fatal.

There was slight chill in one case; the case was a severe one.

Chilly sensations occurred in ten cases; of these, one was a mild case, five were moderately severe, two were severe cases, and two were fatal.

In the remaining nineteen cases, with records of onset, there was no mention of chill, and there probably was none.

Epistaxis, at the onset, is recorded in thirteen cases; of these, one was a mild case, three were moderately severe, six were severe cases, and the other three were fatal.

Vomiting occurred in six cases early in the attack. In two of these it recurred occasionally throughout the attack. In two other cases where it occurred there were evidences of peritonitis, and it occurred in one fatal case of peritonitis.

The bowels were loose at the onset, or early in the attack, in six cases. In two they continued loose throughout the illness, and one of these died of intestinal hæmorrhage. In four the bowels became torpid, later in the course of the sickness; of these, one had prolonged, moderately high, but very variable temperature. The heart's action was very weak. The patient died on the fifty-first day.

In seventeen cases the onset was accompanied by diarrhœa. In thirteen of these the diarrhœa persisted during the entire illness. Two were mild, five were moderately severe, and three were severe cases. Three were fatal cases. Of these one died of peritonitis after perforation of the intestine; one after profuse intestinal hæmorrhage. This patient had also, at the time of death, a temperature of 108° F. In four of the seventeen cases the bowels later became torpid or constipated. One of these died of peritonitis.

The bowels were regular at the beginning of the sickness in six cases. Only one was a severe case; and in only one, a moderately severe case, did the bowels later become loose.

The bowels were constipated in nine cases. In seven they remained so throughout. Of these, two died. In two the bowels became loose; one of these died of peritonitis.

The bowels were irregular at the beginning in two cases. In one they became constipated. This patient died of peritonitis. In the other, a mild case, they became loose.

The state of the tongue usually varied greatly during the progress of the attack, frequently changing from dry to moist, or *vice versa*, sometimes within a few hours. The fact of these variations prevents a satisfactory statement of the conditions of the tongue in the various phases of the disease. Nevertheless, the average condition of the organ during the periods of maximum temperature may thus be given.

In eight cases there was a moist, pasty coat during the period of highest temperature. Of these, three were mild, one moderately severe, and four quite severe. Not all of the latter had excessively high temperatures, but some of them were sick for a long time.

In one moderately severe case the tongue remained red and moist throughout, although the teeth and lips were dry and covered with sordes.

Dryness of the tongue is noted in six cases. Dryness, with whitish coat, in five cases. Two of these were mild, two moderately severe, and was one fatal death from peritonitis occurred on the seventeenth day.

The tongue was dry and brown, and the mouth very foul, in eleven cases. One was moderately severe, two severe, and seven were fatal cases.

It was dry and red and denuded in four cases, all severe.

This summary merely exemplifies the fact that, as a rule, the higher the fever, the dryer and fouler the tongue, but the importance of attention to this organ does not appear.

Its great value lies in the fact of the daily variations. It is often the barometer which furnishes information of the activity or sluggishness with which the functions of life are carried on.

Examinations of the chest showed normal conditions throughout the illness in twelve cases. Of these, two were mild, four were moderately severe, and four were severe cases. Two were fatal; one of these died of peritonitis, the other of exhaustion.

The respiratory sounds were rough in two mild cases; very harsh and dry in one case, a fatal one.

Râles were found at bases of both lungs in eight cases. Four were moderately severe, two were severe cases, and two were fatal. One died of peritonitis and one of intestinal hæmorrhage.

Râles were found extensively over one lung in two cases, one moderately severe, one fatal from perforation of intestine, which occurred at the height of the illness.

Râles were heard extensively over both lungs in five cases, two of which were severe, and three were fatal. Two died of peritonitis, one on the fifteenth and the other on the seventeenth, and one of intestinal hæmorrhage on the twenty-fourth day.

In two cases, one of them fatal, there was unmistakable phthisis.

In most cases the bronchitis was developed only after some days of continuous, rather high temperature.

In nine cases there was no note of the condition of the chest during the height of the fever.

Examinations of the abdomen were not recorded in six cases.

In three cases there was no abdominal tenderness.

In sixteen cases there was pain or tenderness limited to right iliac region.

In one case tenderness limited to the right and left iliac regions.

Diffuse abdominal pain existed in seven cases.

Diffuse abdominal pain greatest in the right iliac region in four cases.

Diffuse abdominal pain greatest in both iliac regions in two cases.

Diffuse abdominal pain greatest in the epigastric region in two cases.

There was moderate tympanites in nineteen cases. Tympanites with great distension of the abdomen in six cases. Three of these were fatal cases of peritonitis. The other three were dangerously sick but there was not conclusive evidence of peritoneal involvement.

The abdomen was very lank in two cases, one mild, the other died rather early of collapse due to perforation. The temperature had not been above 102.5° F. In the remaining cases the degree of abdominal fullness was not noted.

Gurgling was noted in only ten cases of the thirty-five in which the abdominal signs were recorded.

In three cases the absence of spots was recorded.

An eruption was recorded in eighteen cases. In these the eruption was confined to the abdomen in twelve cases. It was scattered over whole trunk in six cases. In some of the latter a few spots found upon the extremities.

The papules were not typical in every instance, but in some were much more raised than usual, and in a few instances became pustular.

In three cases the absence of an eruption is recorded. In the remaining cases there is no note of the presence or absence of spots.

The liver was notably enlarged in two cases, and the spleen in three cases, all of which were severe and one died. No doubt many cases of splenic enlargement escaped notice.

The mental condition during the height of the fever was not recorded in eleven cases.

In one moderately severe case the patient was bright throughout the illness.

In one quite severe case the patient had no other mental disturbance than dizziness.

Eleven patients were dull, of these two were mild, six were moderately severe, one was a severe case, and one patient died early in attack from perforation of intestine.

In five cases the patients were very stupid.

One of them was a moderately severe case, and four were fatal.

Restlessness was a well-marked symptom in three cases, one was a moderately severe case, and two were quite severe. In the first of these the restlessness was only marked at night, when the temperature was highest (103.5°). During the day the patient was dull.

Moderate delirium occurred in seven cases.

One of these patients had a mild illness; the highest recorded temperature was 102°, with a pulse of 84, and the tongue remained moist at the edges throughout the illness. It occurred in two moderately severe cases, in one of these coming on only at night when the temperature was highest (103–104°). Four of the patients died.

Two patients were wildly delirious; both died.

The date of highest temperature was not known in fourteen cases. These were admitted to the hospital late in the course of their illness.

Eight cases reached the highest temperature during the first week in bed. In two of these it was 102.5°. In two between 103° and 104°. In four between 104° and 104.5°. One of these patients died, upon the sixteenth day, of peritonitis. The temperature was irregular, varying between 101° and 104° in the evening.

In another case the temperature ranged between 103° and 104° in the evening at the end of the first week, the pulse ranging between 92 and 112, and the respiration about 40. At end of the second week the temperatures were 99.5° to 100°, pulse 78, and respirations 52. There was bronchovesicular respiration over the upper left lobes of both lungs, accompanied by moist râles, but there was no dullness and no note of pain, or of abnormal physical signs at bases of lungs. Recovery was perfect. There were other cases of very rapid rapid respiration, but this was peculiar, in that the rate of breathing increased after the temperature and pulse-rate had fallen.

Nineteen cases reached the maximum temperature during the second week of illness. In two of them the highest recorded temperature was 102° to 102.7°. In four cases, 103° to 103.2°; of these, one died, on the twelfth day, of peritonitis.

In eight cases the temperature was 104° to 104.8°. One died, in the third week, of peritonitis. The temperature continued high until death. Another died of asthenia. The remaining cases were not very severe.

In four cases the temperature reached 105° to 105.2°. Of these, one died of exhaustion.

Of those admitted late in the course of their illness, three were convalescent, two were in the second and one in the third week of illness. Another, admitted during third week, with a temperature of 103°, improved rapidly in the hospital. Ten were very sick. Of these, two were admitted in the second week. One had a temperature, on admission, of 104°. The patient died of intestinal hæmorrhage. The other had a temperature of 103°, and reached the maximum, 104.6°, during the fourth week, and then convalesced slowly. Six were admitted during the third week. One of the patients died, in the fourth week, of collapse, due to perforation of bowels.

Of those admitted later than the third week of their illness, two had temperatures, on admission, of 105°. Both died.

In two cases the temperature was 104°. One died of asthenia, the other of peritonitis. In the latter the temperaturecurve, after admission, was very variable, the evening range being from 104° to as low as 98.5°. The day before death it was 97°; on the morning of death it was 101.8°.

One patient was admitted during the fifth week with a relapse of the fever. The evening temperature varied between 99° and 103°.

Two relapses occurred in the hospital, brought on by overheating and over-exertion. In these cases the temperature was high, ranging between 104° and 105°, but the patients did not otherwise seem very sick, and did not complain of feeling so. They were not delirious, or were only flighty at night.

These observations show that in the cases under consideration a rapid rise to maximum temperature, attained in the first week or early in the second week, was not especially dangerous; whereas a more gradual rise and a later attainment to the maximum, even though the elevation was no higher than in the former cases, increased the danger in a marked degree.

It is difficult to estimate the effect of temperature upon the cerebral functions. Prolonged elevation of temperature is evidently an important factor in producing disturbance. But in relapses, very high temperatures without much mental derangement were observed, and this, too, at a time when the forces of life had been taxed severely by the preceding illness, and when the patient would be expected to succumb much more readily. It is true that in relapses the temperature, though high, was not prolonged, and in this they resembled those cases in which the disease runs a rapid course with uninterrupted defervescence. There was, in the relapsed cases, even less disturbance than in primary cases of short duration, with high temperature. Besides this, mental hebetude sometimes came on in the primary attacks before a very high elevation of temperature occurred.

Little can be gathered from the pulse-record in these cases beyond what has already been shown in the temperature-lists, for, with very few exceptions, only the frequency of the pulse was recorded.

Records of the character of the pulse are not sufficiently full or numerous to be of value.

Complications occurring in the course of sickness:

Hæmorrhage from the bowels occurred in five cases, of which three were fatal—one died later of asthenia.

Epistaxis during the course of the illness occurred in three cases, one required plugging of the anterior nares.

Peritonitis occurred in three cases—all fatal.

In a number of other cases there were some evidences of peritoneal inflammation, but it is difficult to say in just how many the complication occurred.

Perforation of intestines occurred in two cases—both fatal; one of peritonitis, the other of collapse.

Parotiditis occurred in one case during convalescence—after recovering from this the patient had crural phlebitis.

This patient's temperature, previous to the inflammation of the parotid gland, had reached normal, but had not become established there. The trouble was accompanied by an elevation of temperature of 2°. This existed with scarcely any remission for a day or two; it then fell to normal.

Crural phlebitis occurred in two cases, both convalescent, and in both the temperature had reached normal. In one case, the patient just mentioned, it occurred on the right side. There was a rise of temperature above normal to 101.8°. The temperature remained up three or four days.

In the other case the trouble was on the left side. The elevation of temperature was not quite so great as in the first case there was also a slight relapse after the patient was nearly well.

Mastitis occurred in two cases. In one the patient was seven months advanced in pregnancy. The trouble occurred late in convalescence, after the temperature had been normal for eight days. The inflammation was slight and of short duration. The temperature rose to 100.5°. The other patient had been nursing a child previous to her illness. She was very sick and died on the twelfth day.

One patient during convalescence experienced great difficulty and distress in voiding urine. It became necessary several times to relieve her by catheterization. The urine was normal.

In two cases there was well-marked periodicity in the temperature curves—the evening temperature rising higher every other day. In each case convalescence was long.

One patient was seven months advanced in pregnancy. She had been sick three weeks on admission. At that time the evening temperature was 103.6°. There were marked morning remissions and the temperature was evidently falling. It reached normal in ten days. She did not miscarry.

| | | | | | | | |
|---|---|---|---|---|---|---|---|
| The duration | of the | illness | was in | 1 | case | 3 | weeks. |
| " | " | " | " | 4 | " | 4 | " |
| " | " | " | " | 9 | " | 5 | " |
| " | " | " | " | 3 | " | 6 | " |
| " | " | " | " | 1 | " | 7 | " |
| " | " | " | " | 7 | " | 8 | " |
| " | " | " | " | 2 | " | 9 | " |
| " | " | " | " | 1 | " | 10 | " |
| " | " | " | " | 1 | " | 12 | " |

There were twelve deaths, or about 30 per cent. of the cases treated. Five died of asthenia without complication. Two died during height of fever, the length of the illness was not known. The other three on the 20th, 22d and 51st days respectively.

Three died of peritonitis on the 12th, 16th and 17th days.

One of perforation of the bowel and collapse on the 25th day.

One of perforation and peritonitis ; the length of the illness was not known.

One of intestinal hæmorrhage on the 22d day.

One on the 12th day after severe intestinal hæmorrhage on the morning of her death, and with a temperature at the time of death of 108°.

The treatment was principally expectant.

Tonics and mineral acids were given in all cases. Whisky and digitalis were given when needed.

The sponge-bath and the cold-pack were used to reduce the temperature. In several cases quinine was given in doses of 15 to 20 grains with good effect, but usually a more prompt reduction followed the use of cold.

In two cases, where the intestines were greatly distended by gases, the rectal tube was used, but without relieving the patient. Charcoal and turpentine by mouth gave little, if any, relief.

Turpentine enemata afforded slight temporary relief.

In intestinal hæmorrhage ice was applied locally, ergot was administered internally or hypodermically. Absolute rest was enjoined.

In peritonitis, opium was given and turpentine stupes and hot fomentations applied.

In excessive restlessness and delirium, bromides or morphine were given at night. These cases usually needed very energetic stimulation as well.

Intercurrent local inflammation, such as mastitis, parotiditis and phlebitis, were managed with hot anodyne applications. There was no general treatment except the quinine and acid tonic.

Glenoin was used in one case as a heart stimulant, but without good results. The case was a very unfavorable one, and terminated in death on the 51st day, after the patient's extremities had been cold and blue for a good many days.

